# Supplementary material for: Marginal Zinc Deficiency Promotes Pancreatic Islet Enlargement While Zinc Supplementation Improves the Pancreatic Insulin Response in Zucker Diabetic Fatty Rats
Source: Nutrients. 2024 Jun 10;16(12):1819. doi: 10.3390/nu16121819 (PMC11206688; doi:10.3390/nu16121819)
Supplement: Supplementary file 1 [file nutrients-16-01819-s001.zip › nutrients-3021247-supplementary.pdf]

Table S1. Diet Formulation[1,2].

	MZD<br>4 mg Zn/kg	ZC<br>30 mg Zn/kg	ZS<br>300 mg Zn/kg
**Ingredients (g/kg)**[3]			
Dextrose	609	600	510
Egg White	212.5	212.5	212.5
Cellulose	50	50	50
Mineral Mix (AIN-93G-MN, zinc-free)	35	35	35
Vitamin Mix (AIN-93-VX)	10	10	10
Choline Bitartrate	2.5	2.5	2.5
Biotin Mix[4]	10	10	10
Zinc Premix[5]	1	10	100
Soybean Oil	70	70	70

[1]MZD, marginal zinc deficient diet; ZC, zinc control diet; ZS, zinc supplemented diet.
[2]Zinc concentrations in the diets were confirmed by atomic absorption spectrometry and were MZD = 4 mg Zn/kg diet, ZC = 30 mg Zn/kg diet, ZS = 302 mg Zn/kg diet.
[3]Obtained from Dyets (Bethlehem, PA).
[4]Biotin Mix was prepared using 200 mg biotin/kg dextrose since egg white was the protein source.
[4]Zinc Premix was prepared using 5.775 g zinc carbonate/kg dextrose.

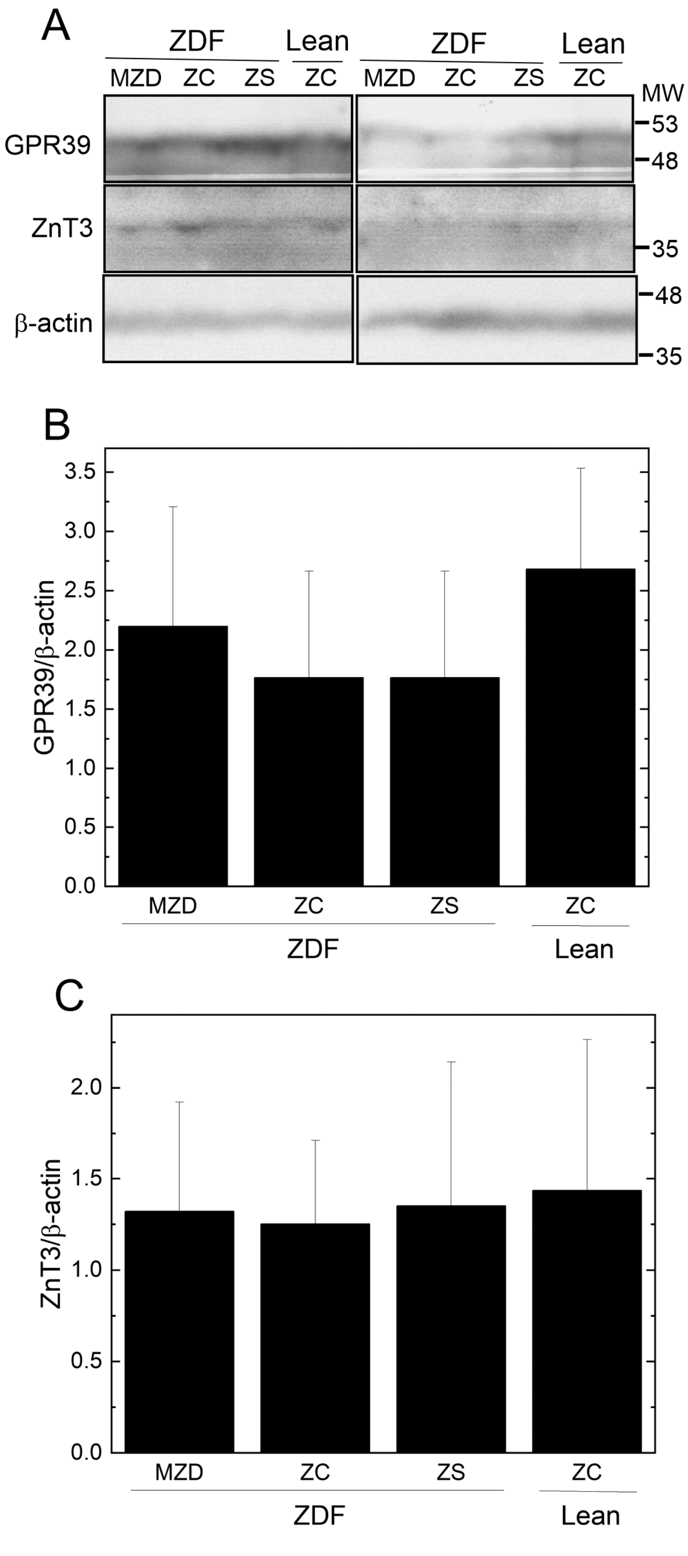

Figure S1. GPR39 and ZnT3 in epididymal fat. Representative Western blots (A) showing relative levels of GRP39 and ZnT3 in epididymal fat samples. The position of the molecular weight (MW) markers (in kDa) are indicated. Densitometry was used to quantify the bands of interest which were normalized to β-actin. The graphs show the relative band intensity as means ± SD (n=7-10) for GPR39 (B) and ZnT3 (C). The data were analyzed by one-way ANOVA and there were no significant differences. Significance was set at $P<0.05$. Abbreviations: Lean, lean (+/?) control rats; MZD, marginal zinc deficient diet; ZC, zinc control diet; ZDF, Zucker Diabetic Fatty rats; ZS, zinc supplemented diet.

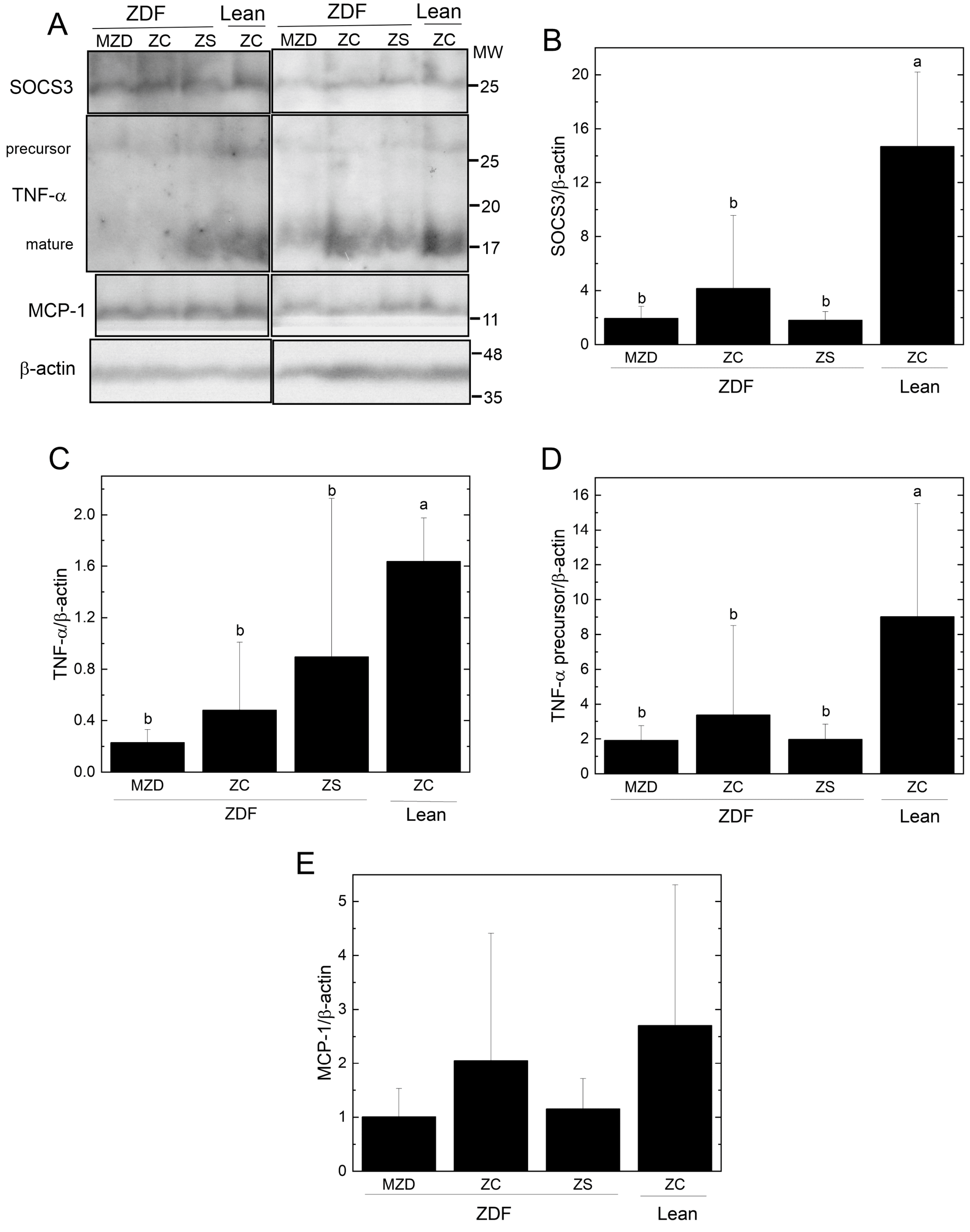

Supplementary Figure S2. SOCS3, TNF-α and MCP-1 in epididymal fat. Representative Western blots (A) showing relative levels of SOCS3, TNFα and MCP-1 in epididymal fat samples. The position of the molecular weight (MW) markers (in kDa) are indicated. Densitometry was used to quantify the bands of interest which were normalized to β-actin. The graphs show the relative band intensity as means ± SD (n=6-10) for SOCS3 (B), mature TNF-α (C), TNF-α precursor (D), and MCP-1 (E). The data were analyzed by one-way ANOVA followed by post-hoc testing with Duncan's multiple range test. Columns with difference letters are significantly different ($P<0.05$). An absence of

letters indicates no significant differences. Abbreviations: Lean, lean (+/?) control rats; MCP-1, monocyte chemoattractant protein-1; MZD, marginal zinc deficient diet; SOCS3, suppressor of cytokine signaling-3; TNF-α, tumour necrosis factor-alpha; ZC, zinc control diet; ZDF, Zucker Diabetic Fatty rats; ZS, zinc supplemented diet.
